# Management of the Thyroid Gland in Papillary Carcinoma of the Thyroglossal Cyst: A Case Report

**DOI:** 10.31729/jnma.5027

**Published:** 2020-07-31

**Authors:** Philip George, Suresh Mani, Ramesh Babu Telugu, Rajiv Charles Michael

**Affiliations:** 1 Department of Head and Neck Surgery, Christian Medical College, Vellore, India; 2 Department of Pathology, Christian Medical College, Vellore, India

**Keywords:** *papillary thyroid carcinoma*, *thyroglossal cyst*, *thyroglossal duct cyst carcinoma*

## Abstract

Carcinoma arising in a thyroglossal cyst is rare. We present a case of anterior neck swelling diagnosed to be thyroglossal cyst clinically which turns out to be a papillary carcinoma arising in thyroglossal cyst. She underwent sistrunk procedure with total thyroidectomy and disease-free on follow up evaluation. Even though preoperative ultrasonography had shown thyroid nodule, the final histology did not show malignancy. There is a paucity of clear-cut guidelines in the management of the thyroid gland in a thyroglossal cyst carcinoma. In thyroglossal cyst carcinoma cases, we recommend thyroidectomy only when there is a thyroid nodule with highrisk features.

## INTRODUCTION

Carcinoma in the thyroglossal cyst (TGC) is a rare entity accounting for only 1% of all thyroglossal cysts. The commonest type is papillary carcinoma.^1^ The symptoms and presentation of carcinoma in TGCs are like its benign counterpart. The preoperative diagnosis of carcinoma is often difficult, and in most cases, thus postoperative histopathology which reveals the diagnosis.^2^ Suspicious clinical and ultrasonographic features may warrant a fine needle aspiration cytology (FNAC) or fine needle biopsy.^3^ The benefits of diagnosing TGC carcinoma preoperatively are pre-operative patient counseling, the decision on the extent of surgery, and deciding on the need for radioiodine therapy.

## CASE REPORT

A 49-years old lady presented with the chief complaints of swelling in the anterior midline of the neck for 6 months. It was insidious in onset and gradually progressive in size. She did not have any complaints of pain or discharge from swelling nor did she have any features of hypo or hyperthyroidism.

**Figure 1.**
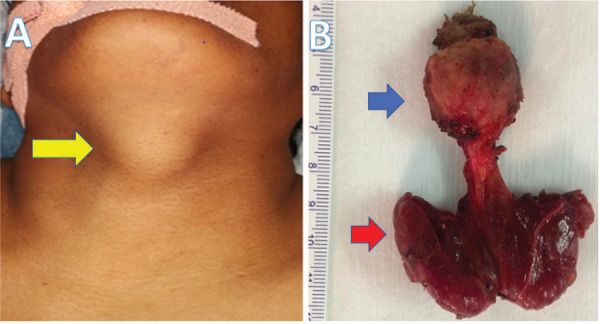
A, B. Anterior midline thyroglossal cyst swelling in subhyoid region (yellow arrow). Gross surgical specimen (Sistrunk operation in blue arrow and total thyroidectomy in red arrow).

On examination, there was a 2 × 2 cm solitary, nontender, firm midline swelling infrahyoid in location, moving with deglutition, and protrusion of tongue (Figure 1 A, B). The thyroid gland was not palpable, and the trachea was central on palpation. There were no palpable lymph nodes.

**Figure 2.**
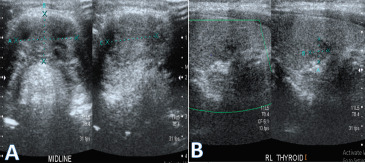
A, B. Ultrasonographic images showing thyroglossal cyst and right thyroid nodule.

Ultrasonography showed a heteroechoic lesion with few cystic areas and multiple echogenic foci, likely calcifications in the midline neck superior to thyroid gland possibly malignancy in the TGC. Another ill-defined heteroechoic lesion in the right lobe of thyroid almost tall as wide with few punctate echogenic foci within suggestive of a thyroid imaging reporting and data system (TIRADS) IV lesion (Figure 2 A, B). FNAC was suggestive of a cystic lesion with respiratory epithelium and moderate atypia, raising the possibility of papillary thyroid carcinoma in a TGC.

**Figure 3.**
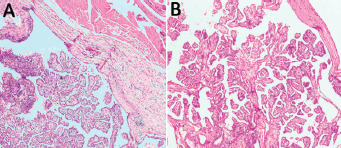
A, B. H&E 40X, showing thyroglossal cyst with papillary carcinoma. H&E 100X, showing papillary carcinoma with arborizing papillary fronds and fibrovascular cores.

In view of the TIRADS-IV lesion in the right thyroid lobe and the TGC with probable malignancy, a total thyroidectomy (TT) along with Sistrunk operation (SO) was planned. Patients and relatives were counselled regarding the nature of the disease and the need for surgery along with lifelong thyroxine supplementation. Intraoperatively, a 2 × 2 cm firm lesion was present infrahyoid in a location without extension to the tongue base. TT and SO were done along with excision of the body of hyoid and the specimen was removed in Toto and sent for histopathological examination (Figure 1 A, B). Final histopathology was suggestive of papillary thyroid carcinoma (classic variant) arising in a TGC (Figure 3 A, B). The maximum size of the tumor was 2.1 cm. The hyoid bone, skeletal muscle, both lobes of thyroid, isthmus, and pyramidal lobe were free of tumor. Pathological staging was pT2NxMx.

Her post-operative period was uneventful. The patient underwent an I^131^ radioiodine whole-body scan after 3 months which was negative for residual disease.

## DISCUSSION

Theories on the development of carcinomas in the TGC may be de novo in the thyroid remnants present in the TGC.^4^ These are termed primary TGCCa and secondary are metastasis from papillary carcinomas of the thyroid gland. Other possible causes may be the multifocal origin of carcinomas in genetically predisposed individuals. Primary TGCCa should fulfill the Widstrom criteria which include: (i) origin should be from the wall of a thyroglossal remnant, (ii) lymph node metastasis should be excluded by histological demonstration, and (iii) primary thyroid malignancy must be absent.^5^ Our case was a primary TGCCa as thyroid gland did not have the disease.

The suspicious clinical characteristics are cervical lymphadenopathy and change in the consistency of cyst. FNAC from solid areas are preferred and may be better performed under ultrasound guidance. Many of the TGCCas are detected in the final histopathology after Sistrunk operation for TGC. Though diagnostic pitfalls are frequent in FNAC, the major criteria identified are high cellularity, papillary formations, cells with enlarged nuclei showing anisonucleosis and powdery chromatin, and definite nucleoli. Intranuclear pseudo inclusions and grooves are significant findings. Psammoma bodies, multinucleate giant cells may be present.^3^ Fortunately, in this case, we could diagnose carcinoma pre-operatively and we could plan the management appropriately.

In cases of TGCCas, there are no clear-cut guidelines for deciding on the management of the thyroid gland. The theory that TGCCa arises denovo favour only Sistrunk operation as management. Total thyroidectomy has been advocated by certain authors for differentiated thyroid carcinoma in a thyroglossal cyst irrespective of thyroid involvement. The advantages of total thyroidectomy are that it will aid staging and facilitate identification of metastasis and recurrence by radioactive iodine therapy.^6^ It also avoids the complications of redo surgery.

In a systematic review by Hanim, et al. 61% of the series underwent total thyroidectomy of which only 23.4% had a tumour in the thyroid gland.^7^ Whether thyroidectomy needs to be done in all cases is a debatable topic. Several authors recommend total thyroidectomy as a follow-up procedure in TGCCa. The risks of recurrent laryngeal nerve injury and hypocalcemia and the benefits of radioactive iodine (RAI) therapy following total thyroidectomy need to be considered. Our case had a TIRADS-IV thyroid nodule at presentation and hence we had given the patient the options of hemi vs. total thyroidectomy and the patient opted for a total thyroidectomy. In a study by Patel, et al. on the management of well-differentiated TGCCa, the only significant predictor for overall survival was the completeness of excision of TDC.^8^

Recently, prognostic risk group assessments are used to identify patients who would benefit from additional TT. High-risk factors identified by Plaza, et al. for additional TT with RAI and suppressive therapy were (a) age >45 years, (b) past radiation exposure, (c) thyroid gland tumor on radiology (d) presence of clinical/ radiological nodes, (e) tumor >1.5 cm in diameter, (f) cyst-wall invasion and, (g) positive margins on HPE.^9^ The incidence of neck nodal metastasis from various studies are in the range of 10 to 15%. According to J M Manipadam, et al. there is no role for elective neck dissection in N_0_ neck.^10^

Tharmabala, et al. proposed classification on risk stratification into low, moderate, and high risk depending on age, size, presence of thyroid lesion, the involvement of margins, histological features, multifocality, nodal, and cyst wall involvement.^5^ All cases are advised of a Sistrunk operation. Depending on risk factors, TT and RAI are advised in a moderate risk group and TT, neck dissection with RAI in the high-risk group. According to this risk stratification, our patient came in the moderaterisk group in view of age and thyroid lesion. The role of RAI in the management of TGCCa does not have clear guidelines. Advanced disease with involvement of thyroid and lymph node metastasis who undergo total thyroidectomy are candidates for RAI.^9^ We opine cases should be categorized based on risk stratification and the decision on thyroidectomy has to be reserved for high-risk patients.
